# A novel variant in the GNE gene in a Malian patient presenting with distal myopathy

**DOI:** 10.21203/rs.3.rs-4004982/v1

**Published:** 2024-03-07

**Authors:** Mahamadou KOTIOUMBE, Alassane B. Maiga, Salia Bamba, Lassana Cissé, Salimata Diarra, Salimata Diallo, Abdoulaye Yalcouyé, Fousseyni Kané, Seybou H. Diallo, Dramane Coulibaly, Thomas Coulibaly, Kékouta Dembélé, Boubacar Maiga, Cheick O. Guinto, Guida Landouré

**Affiliations:** Faculté de Médecine et d’Odontostomatologie, Université des Sciences, des Techniques et des Technologies de Bamako, Bamako; Faculté de Médecine et d’Odontostomatologie, Université des Sciences, des Techniques et des Technologies de Bamako, Bamako; Faculté de Médecine et d’Odontostomatologie, Université des Sciences, des Techniques et des Technologies de Bamako, Bamako; Service de Neurologie, Centre Hospitalier Universitaire du Point “G”, Bamako; Faculté de Médecine et d’Odontostomatologie, Université des Sciences, des Techniques et des Technologies de Bamako, Bamako; Service de Neurologie, Centre Hospitalier Universitaire “Gabriel Touré”, Bamako; Faculté de Médecine et d’Odontostomatologie, Université des Sciences, des Techniques et des Technologies de Bamako, Bamako; Faculté de Médecine et d’Odontostomatologie, Université des Sciences, des Techniques et des Technologies de Bamako, Bamako; Faculté de Médecine et d’Odontostomatologie, Université des Sciences, des Techniques et des Technologies de Bamako, Bamako; Service de Médecine, Centre Hospitalier Universitaire “Le Luxembourg”, Bamako; Faculté de Médecine et d’Odontostomatologie, Université des Sciences, des Techniques et des Technologies de Bamako, Bamako; Faculté de Médecine et d’Odontostomatologie, Université des Sciences, des Techniques et des Technologies de Bamako, Bamako; Service de Neurologie, Centre Hospitalier Universitaire du Point “G”, Bamako; Faculté de Médecine et d’Odontostomatologie, Université des Sciences, des Techniques et des Technologies de Bamako, Bamako; Faculté de Médecine et d’Odontostomatologie, Université des Sciences, des Techniques et des Technologies de Bamako, Bamako

**Keywords:** Distal myopathy, GNE, novel variant, Mali, Africa

## Abstract

**Background:**

GNE myopathy (GM) is a rare autosomal recessive disorder caused by variants in the *GNE* gene and characterized by progressive distal muscle weakness and atrophy. We report a novel variant in the*GNE* gene causing GM in a consanguineous Malian family.

**Case presentation:**

A 19-year-old male patient from a consanguineous family of Bambara ethnicity was seen for progressive walking difficulty and frequent falls. Neurological examination found distalmuscle weakness and atrophy and reduced tendon reflexes in four limbs. Electroneuromyography (ENMG) showed an axonal neuropathy pattern with reduced distal motor amplitudes. Charcot-Marie-Tooth (CMT) gene panel testing (Medical Neurogenetics LLC, Atlanta, GA) was negative. However, whole exome sequencing (WES) revealed a novel biallelic variant in *GNE* (c.1838G>A:p.Gly613Glu), segregating with the phenotype in the family. This variant is predicted to be pathogenic by several *in silico*prediction tools including CADD= 29. Moreover, protein folding model showed major structural disruptions in the mutant protein.

**Conclusion:**

This study reports a novel variant in the *GNE* gene causing GM, the first molecularly diagnosed in sub-Saharan Africa (SSA). It highlights the diagnosis challenges in this region and broadens the genetic spectrum of this rare disease.

## Background

GNE myopathy (GM) (OMIM: 605820) also known as Nonaka myopathy (NM) or hereditary inclusion body myopathy (HIBM) is a rare autosomal recessive disorder caused by variants in the *GNE* gene [1]. Its global prevalence is estimated from 4 to 12:1.000,000 [2]. Clinically, GM typically manifests by bilateral foot drop caused by weakness of the anterior tibialis muscles with early adulthood onset. The disease progresses slowly over time to involve skeletal muscles throughout the body, with relative sparing of the quadriceps at the late stages of the disease [3–5]. Other additional symptoms including thrombocytopenia, cardiomyopathy, and neuropathic features have been previously described in patients with GM [6–8]. However, the mechanism by which GM leads to neurogenic disorders remains unknown.

Although several pathogenic variants have been reported to cause GM worldwide [7], only a few cases have been described in the North African populations [9, 10]. In this study, we report a novel variant in *GNE* causing GM in a Malian consanguineous family.

## Case presentation

**Figure 1 :**
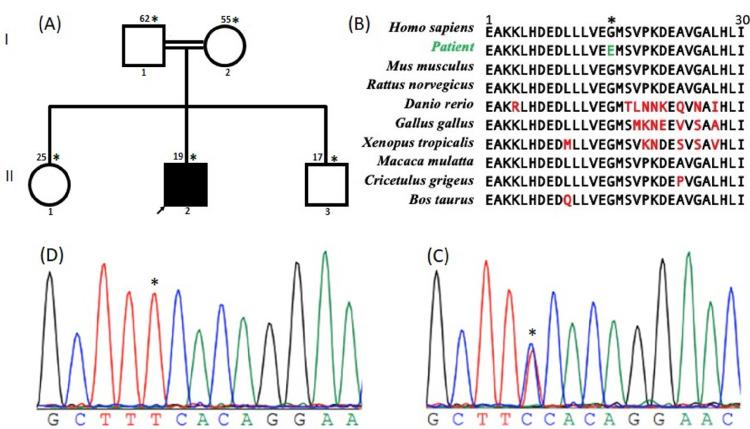
Pedigree and genetic data of patient with GNE Myopathy. **A)**Pedigree of the family showing an autosomal recessive inheritance pattern. Asterisks represents individuals seen in clinic, the arrow designates the proband and numbers on top are ages. **B)**Portion of amino acids conservation of GNE protein, showing the Gly613 residue in a highly conserved region across various species. **C and D)** Chromatograms showing both heterozygotes (parents and siblings) and homozygote variants “C” to “T” change (patient), respectively.

A 19-year-old male and his healthy relatives of Bambara ethnicity were seen for a progressive walking difficulty. He is from a consanguineous marriage (Fig. 1A) and with no remarkable past medical history. The disease started at age 17 with a gait difficulty that worsened gradually and has led to frequent falls. Neurological examination found moderate distal muscle weakness in the upper limbs and severe in the lower limbs. He had decreased tendon reflexes, bilateral flexor plantar reflexes. Muscle atrophy was noted, more marked in lower limbs involving the tibialis anterior muscles. He did not present sensory impairment as well as cardiac or auditory symptoms. Creatine Kinase (CK) level dosage was not done due to the unavailability of the patient. Nerve conduction studies showed reduced compound motor action potential (CMAP) amplitudes and none-response sensory nerves in lower limbs. Six years later, at age 25, he was seen in our clinic with severe symptoms and was wheelchair-bound.

Initially, the clinical pattern was suggestive of peripheral neuropathy, and the CMT panel testing performed in CLIA certified laboratory (Medical Neurogenetics LLC, Atlanta, GA) was negative. However, whole exome sequencing (WES) identified a novel homozygous missense variant c.1838G > A, leading to a Glycine to Glutamate change at position 613 (p.Gly613Glu) in the *GNE* gene. This variant was confirmed by Sanger sequencing, and the Gly613 residue is highly conserved across a wide range of species (Fig. 1B). In addition, the variant was shown to segregate with the disease status in the family (Figs. 1C and 1D). This variant is predicted to be deleterious by several *in silico* tools (CADD = 29) and classified as likely pathogenic (PP3, PM1, PM2) according to the American College of Medical Genetics (ACMG) criteria. More informations on the deleteriousness of the variant are provided in the Supplementary Table S1. Clinical and genetic findings are summarized in Table I.

**Figure 2 :**
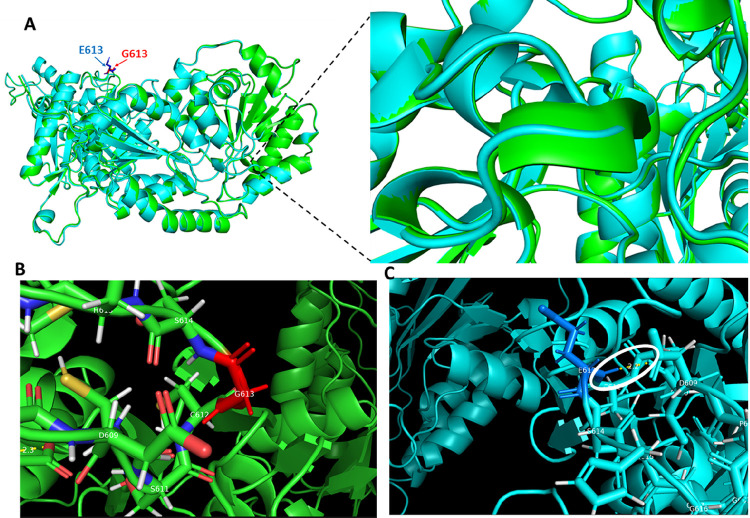
Three-dimensional (3D) structure of GNE proteins. **A)**Superimposed 3D structure of wild-type (green) and mutant (light blue) showing structural changes (loss of helix). **C)** Hydrogen bond analysis showing new bonding interaction of the mutant with Asp609, highlighted with a white circle when compared to **B)** wild-type.

Importantly, secondary and three-dimensional (3D) structural analyses reveal several major disruptions in the mutant protein compared to the wild-type involving helical structures (Fig. 2A and S1). In addition, hydrogen bond analysis showed that the Glycine-613 is not directly involved in bonding interaction (Fig. 2B). However, the mutant Glutamate-613 gained four hydrogen bonds with Cysteine-610 (Fig. 2C). This change is predicted to impact the physicochemical properties of the protein making it unstable while the wild-type is predicted to be stable.

## Discussion

The typical phenotype of GNE myopathy includes adult-onset, progressive distal muscle weakness and atrophy of the lower limbs sparing quadriceps at the late stage of the disease course [11].

Although GNE myopathy was described decades ago, its diagnosis is highly challenging solely based on clinical and electroneurographic findings. Clinically, peripheral neuropathy and GNE myopathy share similar manifestations [12]. In addition, electrophysiological characteristics of motor neuron involvement have also been described in previous studies, suggesting axonal neuropathy [13 – 15] as seen in our patient. However, no response was recorded in the bilateral sural nerve, which could be due to environmental factors. Histological studies can guide the diagnosis in some cases but are not available in Malian setting.

As the clinical and electrodiagnostic findings in our patient were mimicking peripheral neuropathy, CMT gene panel testing was done first but came negative. We took advantage of the WES technique to investigate our case and surprisingly identified a novel homozygous missense variant in the *GNE* gene known to cause GM. Therefore, our study further confirms that molecular diagnosis is highly contributive in discriminating these two entities.

The pathophysiology of the disease is not entirely elucidated to date, but hyposialylation of muscle glycans is thought to play an essential role [3, 16, 17]. Molecularly, *GNE* encodes the enzyme sialic acid epimerase, which is responsible for the last step of sialic acid biosynthesis [18–20]. Secondary structure analysis showed some major changes in the mutant protein involving the alpha helix and beta strand, predicted to impact the overall folding and physicochemical properties of the protein. Furthermore, based on InterPro domain search, this variant occurs in the ATPase, a nucleotide-binding domain that exhibits ATPase activity likely crucial to the function of GNE.

This is likely the first GNE myopathy molecularly diagnosed in the SSA found in Mali and the fourth in Africa after those reported in Tunisia and Egypt from unrelated families [9, 10].

## Conclusion

We report a novel variant in *GNE* causing GNE-myopathy. It highlights the challenges of the evidence-based diagnosis in resource-limited settings for this rare condition and broadens its genetic spectrum, globally. As molecular diagnosis has become more accessible with the advent of next-generation sequencing (NGS), there is a pressing need to extend genetic studies to underexplored populations in order to uncover novel variants or genes that could further our understanding of the pathophysiology of the rare diseases and improve their management as clinical trials for several of these diseases are underway.

## Figures and Tables

**Table I :** Phenotypic and laboratory features of patient with GNE myopathy

Patient	Clinical data									Laboratory findings									
	Age (yr)	Sex	Age of onset (yr)	First symptom	Arm weakness		Leg weakness		Sensory loss	Nerve Conductions Studies									Genetic findings
					Proximal	Distal	Proximal	Distal											
II.2	19	M	17	Walking difficulty	Mild	Severe	Mild	Severe	None	Median			Ulnar			Sural	Tibial	Peroneal	*GNE* c.1838G>A; p.Gly613Glu
										SNAP Amp	CMAP Amp	CV m/s	SNAP Amp	CMAP Amp	CV m/s	SNAP Amp	CMAP Amp	CMAP Amp	
										NP	10.1	54	11	NP	86	NR	1.8	1.7	

yr = year, AMP: amplitude, SNAP: sensory nerve action potential, CMAP: compound motor action potential, CV: conduction velocity, m/s: meter per second, NP: not performed, NR: no response

## Abbreviations

ACMG: American College of Medical Genetics
CADD: Combined Annotation Dependent Depletion
CK: Creatine Kinase
CMT: Charcot-Marie-Tooth disease
OMIM: Online Mendelian Inheritance in Man
WES: Whole Exome Sequencing
